# The Anticipated Future of Public Health Services Post COVID-19: Viewpoint

**DOI:** 10.2196/26267

**Published:** 2021-06-18

**Authors:** Haitham Bashier, Aamer Ikram, Mumtaz Ali Khan, Mirza Baig, Magid Al Gunaid, Mohannad Al Nsour, Yousef Khader

**Affiliations:** 1 The Eastern Mediterranean Public Health Network Amman Jordan; 2 National Institute of Health Islamabad Pakistan; 3 Jordan University of Science and Technology Irbid Jordan

**Keywords:** COVID-19, public health, health system, health services

## Abstract

In March 2020, the World Health Organization declared COVID-19 as a global pandemic. The COVID-19 pandemic has affected various public health functions and essential services in different ways and magnitudes. Although all countries have witnessed the effect of COVID-19, the impact differed based on many factors including the integrity and resiliency of the countries’ health systems. This paper presents opinions and expectations of the authors about the anticipated changes in the future of public health at the global, regional, and national levels. The viewpoint is based on the current efforts and challenges that various stakeholders have carried out to control COVID-19 and the contribution from the literature on the future of public health. Numerous agencies and actors are involved in the fight against COVID-19 with variations in their effectiveness. The public health services showed weaknesses in most of the countries, in addition to the lack of adequate curative medicine settings. The pandemic highlighted the need for better governance and stronger and more resilient health systems and capacities. The COVID-19 experience has also emphasized the importance of coordination and collaboration among the countries and stakeholders. The COVID-19 pandemic might lead to a wide discussion to improve international and national approaches to prepare for and respond to similar events in terms of preparedness and response mechanisms and tools. Public health will not be the same as before COVID-19. New health priorities, approaches, and new agendas will be on the table of the global platforms and initiatives. More investment in research and technology to meet the demand for new vaccines and medicines, innovative methods like distance learning and working, more respect and remuneration to health professionals, and normalization of the public health and social measures that were induced during the COVID-19 pandemic are expected to be seen in future.

## Introduction

As of February 6, 2021, more than 105.6 million COVID-19 cases and nearly 2.3 million deaths were reported from almost all countries worldwide [1]. Following the reported first cases of the COVID-19 pandemic, many countries applied variable public health and social measures to prevent and control the wider spread of cases. There are major concerns and uncertainties regarding not only when to return to “normal” activities but also the anticipation of what that *new normal* might be like [2]. Numerous agencies and actors have been involved in various public health interventions to fight against COVID-19 with variations in their effectiveness. This paper highlights the current efforts and interventions that are carried out by various stakeholders to control COVID-19. The objective is to initiate the discussion and share ideas about the future of public health post COVID-19 at the global, regional, and national levels.

## COVID-19 Response: Leading Players and Partners

Many agencies have been involved in responding to and controlling the COVID-19 pandemic. The following sections provide an overview of the main players and partners, and their roles.

### Ministries of Health

An obvious observation is that the ministries of health (MOH) have restored their leading role during their fight against COVID-19. For decades, such emergencies were essentially managed through the United Nations agencies and nongovernmental organizations (NGOs). The threat of COVID-19 has brought other government sectors closer to and more involved in the work of MOH [3]. However, many gaps were detected in the health system in most countries. In general, the public health services showed weaknesses in most of the cases, in addition to the lack of adequate curative medicine settings. An example was provided by the survey conducted by the World Health Organization (WHO), which showed that around 75% of countries reported a substantial degree of noncommunicable disease service disruptions [4].

The pandemic highlighted the need for better governance and stronger and more resilient health systems and capacities. It is time for the health systems to consider shifting the paradigm toward public health and preventive medicine. The COVID-19 pandemic has provided an opportunity to enhance coordination and collaboration among countries and stakeholders. Collaboration between the health care communities, public health professionals, subnational management, educational institutions, NGOs, transport, security agencies, and other line ministries is expected to continue in the future. Still, better coordination and collaboration are needed among various sectors that are involved in health interventions and response under the leading role of MOH.

### World Health Organization

The WHO continues to provide its technical and logistic support to countries and the other UN agencies. It has developed and disseminated various protocols and tools for COVID-19 surveillance, case definition, planning, testing, isolation, quarantine, protection, and treatment. It has shipped millions of testing kits and equipment to needy countries [5,6]. In addition, the WHO has launched an academy to provide access to related training materials for health workforces [7].

The COVID-19 pandemic might lead to a discussion to improve the WHO’s approach to prepare for and respond to similar events in terms of governance, mechanisms, and support systems, including the International Health Regulations. It is also expected that the new cooperation mechanisms and agreements among countries will be considered to improve the sharing of information and the early warning of similar threats [8].

### Nongovernmental Organizations

Civil societies and NGOs have played a fundamental role in fighting COVID-19 at the local level [1]. Yet, civil societies are expected to play a bigger role in the accountability for funds to tackle the COVID-19 pandemic [3]. The main challenges encountered during their response are that not all of them are adequately structured to anticipate and adapt to a large-scale change in context. Besides, their business model is structured around preplanned projects and programs with identified deliverables and measurable outcomes. COVID-19 has put the NGOs under a funding crisis with a possible future decline in aid budgets and donations [9]. On the other hand, there are ongoing initiatives to establish a COVID-19 response investment vehicle. NGOs’ liquidity funds may be necessary to ensure their sustainability. NGOs need to consider shifts in their mandates and priorities, and to build their capacities to deal with similar events.

Due to COVID-19, humanitarian access becomes a problem due to movement restrictions that might affect access to affected people and areas [10]. The impact on the conflict dynamics is expected to continue in many of the protracted conflict environments in which humanitarians operate. The support to the countries is challenged by accessibility to the various regions and outreach due to the restricted movement. However, this could be tackled through the model adopted by the Eastern Mediterranean Public Health Network, which managed to access those areas through its widespread network of Field Epidemiology Training Programs, rapid response teams (RRTs), and experts. Other NGOs and networks can build on this model, with the required adaptation, in their business model, partnerships, and approaches.

### Private Sector

The private sector has contributed to many aspects like the provision of innovative solutions, including equipment, treatment, and vaccines. For example, in the United States, the private sector contributions to both diagnosis and treatment had led to a decrease in mortality by 44% and an increase in intensive care and nonintensive care hospital beds by 30% and 28%, respectively [11]. During the pandemic, the private sector shared its experience in organizing work and how business has been instrumental in addressing COVID-19 in a myriad of ways, including raising funds and supporting national responses; investing directly in primary health care (PHC); taking critical steps to protect their employees and communities; and leveraging their capabilities in manufacturing, communications, and supply of health commodities, especially personal protective equipment [12].

The contribution of the private sector to COVID-19 control efforts was mainly voluntary, mostly unorganized, and fragmented [13]. There is a need to establish a clear coordination mechanism with the government and NGOs. An additional requirement is a special platform or task force to engage the private sector in the national response to outbreaks like COVID-19 [14]. It is time to consider developing a legal frame for private contributions during emergencies to streamline the support with the national priorities [15]. It is wise to support the private sector post outbreak to ensure continuity of its effective support in future public health emergencies.

### Global Initiatives and Platforms

The interest in empowering vulnerable people, communities, and countries is expected to rise again, as the world realized that the risk of one entity can affect the entire world. The Millennium Development Goals and Sustainable Development Goals (SDGs) could have better prepared countries for this crisis [15]. The world may need to stop and see how to accelerate the achievement of the SDGs and fix any shortcomings and challenges. Post COVID-19, it is expected that everyone will be mobilized around the UN 2030 Agenda and SDGs. COVID-19 might be used as a new excuse for isolationism and, hence, cutting development assistance for health and reframing global health because rich countries have had substantial economic losses [10]. It is also expected that the *north-south paradigm* will come to an end with a deepening division between north and south. New players will have key roles in global health policies and agendas like China and South Korea. Many scholars raised a serious question on whether we are going to see more health spending both at the domestic and the international levels. More investments are expected in prevention measures, higher salaries to medical staff, and improving their health insurance and increase in coverage [10]. Other scholars expect that infectious diseases will take center stage within the global agenda of future projects and initiatives [10]. To cope with the changing environment, the international organizations need to adopt new flexibilities and expanded mandates to match the needs of a pandemic and greater integration of the vertical programs.

Generally, more holistic, comprehensive, and coordinated approaches and strategic interventions become a necessity on the agenda of the multilateral and global initiatives [10]. Stronger intergovernmental and stakeholder platforms and networking are needed. Policies and strategies that directly or indirectly affect or are affected by the health of the public might be revised and updated. Moreover, countries and international partners need to revisit the health financing to tackle similar crises in the future [16].

## Essential Public Health Functions

The COVID-19 pandemic has affected the various public health functions in different ways and magnitude [17]. Although all countries have witnessed the effect of COVID-19, the impact and response in those countries differ based on the integrity of their health systems. This paper tries to highlight the main challenges and the expected changes in selected essential public health services.

In a limited-income country like Pakistan, the COVID-19 outbreak has greatly challenged the health care system due to scarcity of basic health facilities at all levels, insufficient health policies, weak administration and governance, poor status of implementation of national policies, a less integrated system, and an indifferent attitude of the public toward protective measures [18].

### Surveillance, Data Science, and Modeling

Disease surveillance is the backbone of any epidemic response, as it provides information about the sections of the population most at risk, which helps develop targeted interventions to contain the disease spread in the population [19]. In response to COVID-19, countries must adopt innovative solutions and new surveillance tools [20]. They need to run a robust real-time national integrated disease surveillance system with real-time data reported daily [21]. Using the latest technology, targeted smart lockdowns of high-risk areas with active daily sampling and daily monitoring led to a rapid decrease in cases. Examples are phone tracking data, closed-circuit television footage, and dashboards. However, there is still a need for innovative ideas, approaches, and tools to strengthen surveillance capacities. Countries should seriously consider adopting a comprehensive and integrated national surveillance system. This is to be linked to the event-based surveillance and participatory surveillance to intensify active surveillance. Additionally, the COVID-19 pandemic has taught us that we require a new dimension to privacy, protection of human rights, and ethics [22]. The use of digital technology in future epidemics and the lessons learned during COVID-19 will mitigate the effects of any future epidemics, as the health system is expected to be more strengthened, informed, and ready.

### New Approaches for the Health System

A responsive health care system is needed to be able to mount an effective response to any health-related emergency. Countries need to have a clear holistic plan for preparedness and response. Globally, public health remains chronically underfunded, with spending further declined over the past 20 years. Despite the increased attention to public health, it remains unclear whether policy makers will increase investments in the public health infrastructure and workforce [23]. This pandemic has created the demand for better funding and investment in public health. The COVID-19 pandemic has tested the limits of the health care system in many countries. The pandemic has persuaded and forced the governments to inject much-needed funds into the health system. The health system has seen the allocation of unprecedented amounts of finances that have the potential to change the whole outlook of the health system, making it stronger and more responsive to the needs of populations. However, the government needs to create a *permanent budget cap exemption* mechanism for public health functions that are critical to prevent, detect, and respond to infectious diseases. This mechanism is a potential road for stable and increased funding for public health for the long term.

The countries have made use of their RRTs, which have effectively contributed to the detection and management of COVID-19. To respond to the pandemic, many countries required large numbers of trained medical staff. However, it was costly to do effective manual contact tracing efforts. In the United States, this required 100,000 workers and US $3.6 billion [24]. To tackle the shortcomings of traditional contact tracing, many countries worldwide have implemented or expressed interest in automated contact tracing services [25]. Although the advantages of automated contact tracing services can be attractive, governments still need to consider their technical shortcomings and the inherent trade-offs between privacy and efficacy when deciding whether to implement these technologies [26].

After COVID-19, the need to build automated contact tracing services and expand the investment to develop new rapid tests including immunity testing for antibodies is moving forward. The next phase of the pandemic may require the conduction of mass screening at the community level to better understand and hence manage the pandemic. The key questions here are can these be accomplished via the current health system and are our current health systems resilient enough to accommodate the new changes?

### Public Health Services

The goal of all countries is to suppress the transmission of COVID-19 and provide care for all patients [23]. The COVID-19 pandemic has compromised the delivery of essential health services in various countries [27]. For example, the countries of the Eastern Mediterranean Region are challenged with the COVID-19 response, mainly in information sharing, expanding public health measures, protecting health workers, achieving behavior change, ensuring continuity of essential health services, and establishing reliable supply chains [28].

More efforts will be needed to develop and update the National Health Emergency Preparedness and Response Plan. Hospitals were overwhelmed and could not cope with the pandemic in most countries, including Western countries. Rethinking the role of hospitals and other institutions as hubs for care is required.

Many health systems collapsed due to a lack of the required competencies or due to the exhaustion of the working staff. More investment in PHC and shifting in the point of care, such as testing of common infectious diseases and on measures to prevent the spread of infections, are needed [17]. An appropriate mix of good quality hospitals, PHC services, and public health might be considered. A focus on sustainable service delivery is highly needed for bringing health care to patients, to eliminate doctor-patient visits whenever possible, for increasing the use of telemedicine, for manufacturing health commodities, and for strengthening supply chains [10]. PHC services near people’s homes are so important and can be lifesaving. Improving access to vaccinations, screening, education, counseling, and supporting access to treatment are all essential [20]. The health systems are expected to focus on sustainable service delivery, with a significant increase in investment in emergency preparedness to cover the manufacture of health commodities and strengthen supply chains. Patrick Fine expects that health access and the role of social enterprise will be expanded. Among the expected changes in the provision of health services is that more basic services will be assigned for the community health workers to shorten the time of delivery [29]. Health tourism will abate, and instead, doctors are expected to fly to needy countries. Health insurance is expected to cover less and cost more [30].

Some forgotten or undermined health services like mental health and occupational health may see increasing attention [30]. During this pandemic, people were mentally suppressed, and the stigma associated with this disease created another mental crisis. COVID-19 will have a lasting impact on many people’s mental health and well-being. To support people’s recovery and resilience, public mental health and statutory mental health services, alongside the community welfare sector, will need to be resourced to meet increased demand and maintain a focus on vital preventative work.

On the other hand, public health laboratories play a vital role in protecting the health of people from emerging health threats. During a brief period of a few months, several public health labs have been established, all fully functional, properly equipped, and ably manned. This has again resulted in the strengthening of the lab capacity and health system. Further strengthening and digital linkage of this laboratory network will serve as a mechanism for laboratory-based surveillance of diseases other than COVID-19 as well. Therefore, the health system is expected to be well equipped to handle epidemics of any scale in the future.

Clearly defined legal powers and enforced public health law are needed to respond to outbreaks of contagious and serious diseases at the national level. To achieve this objective, countries need to update and refine their public health laws. This will ensure minimizing the transmission of infectious diseases. Besides, it is recommended to ensure the clarity of roles, relationships, and coordination mechanisms in health system governance and across governments.

### Public Health Education and Priority Training

During the pandemic, health professionals across the health care system have worked tirelessly in the most challenging circumstances. The workforces have been a significant challenge that was observed during the response to COVID-19 [20]. Responding to the COVID-19 pandemic required an increase in the number of workforces [31]. Many health systems collapsed due to a lack of the required competencies or due to the exhaustion of the working staff. The COVID-19 pandemic could inspire young people to choose careers in public health. According to the Council for Public Health Education, to meet this increasing demand, there is a need for specialized training as part of professional development to ensure readiness for future similar challenges [32].

Medical and health curricula need to be updated and enriched to meet the emerging changes and needs in public health, both the core competencies as well as leadership and management skills. This is to be at the levels of undergraduate, postgraduate, and professional training. Distance training and e-learning become a necessity, and institutes and training providers must consider these modalities in their plans.

A good example of such specialized, on the job training for professionals is the Field Epidemiology Training Program. In fact, the aim of the Field Epidemiology Training Program is to improve the epidemiologic capacity of a country’s public health workforce to detect and respond to health threats [33]. Hence more resources are to be allocated for establishing new Field Epidemiology Training Programs and strengthening the existing programs, with special focus on the frontline and intermediate levels. Hopefully, the pandemic will bring more respect and remuneration to health professionals, and health as a profession will be considered a high-risk job, and laws and regulations will need to be revisited.

### Risk Communication

It was a challenge to advocate to the public and even the professionals on the importance of public health activities. Because of the COVID-19 pandemic, people, including decision makers, are now more open and receptive to the importance of *public health*. This momentum is needed to advocate more for public health agenda and adopt prohealth policies and secure more funding [27].

During the COVID-19 pandemic, health systems were challenged by a surplus of information. Some information was false and potentially harmful. Besides, the inaccuracy of the information and its fast distribution through various media channels made it more difficult for people to identify verified facts and advice from trusted sources. Misinformation rumors regarding COVID-19 become a substantial problem in epidemic responses [34]. Robust efforts were seen by the countries in this regard both in terms of a legal framework and an effective risk communication mechanism. Public awareness during the pandemic was ensured with the active involvement of print and electronic media and through community engagement programs. Many lessons were learned that will translate to improving the risk communication strategies and approaches for future pandemics.

Public health and social measures includes personal protective measures, environmental measures, physical distancing measures, and travel-related measures. These will continue and become more necessary [30]. There is a need to make good use of all community resources in future efforts [31]. Community health workers can play a role in community-based COVID-19 emergency response teams [29]. The public can be enabled to take part in the implementation of public health and social measures. Health education and health messages played a crucial role in controlling COVID-19. The public is used to relying on governments to provide advice and protect their well-being. However, many websites (official and informal), hotlines, webinars, and social media have been increasingly used by the public during the COVID-19 pandemic to get updated news and messages. This trend in relying more on community-based communication is expected to continue and become the default for future events [10].

### Research and Innovative Public Health Solutions

Thousands of research articles have been published since the start of the COVID-19 pandemic. This is expected to grow especially with the research work that depends on big data. The Global Research Forum developed an initial COVID-19 Global Research Roadmap to guide a united COVID-19 agenda for research and development. Other funding opportunities are already there, and more are expected to come. The main areas for future research on COVID-19 could be research to support policy making and provide evidence-based solutions [19]; medical and technological research; and innovation to accelerate the production of the vaccines and treatments, tests, and equipment. Besides, expansion is expected in the use of technology to develop new tools to assist in executing public health functions (eg, surveillance functions, electronic and mobile phone–based data collection, and dashboards for logistic management systems) [10]. On the other hand, diagnostics based on artificial intelligence, cloud-based storage of medical records, integration of information in and outside the hospitals, and increased use of robotics have already been introduced to the health services [20]. Those will come under the bigger umbrella of telehealth and telemedicine [30]. Telehealth services were not new for developed communities, but its use was limited. Internet and Android apps increased the use of telehealth.

Different interventions and measures have been applied by various countries to respond to and control the COVID-19 pandemic. However, many questions were raised on the efficacy of those interventions and measures and how transparent they were. One can expect the need for evaluation studies to answer those questions and others like the accountability of the emergency operations and projects, COVID-19–related vaccination assessment, and social consequences and economic costs of public health and social measures.

Distant working becomes a reality with more use of technology like teleconferencing and videoconferencing systems, which requires confidence in digital security. Health “Immunity” Passports are expected to become mandatory for travel besides other measures like e-tickets, e-queuing, spacing, masks and gloves, and on-site testing [20].

## Conclusion

Many countries applied various public health and social measures to prevent and control the spread of the disease. The pandemic highlighted the need for better governance and stronger health systems and capacities. It is recommended to consider shifting the paradigm toward public health and preventive medicine. In addition, the COVID-19 pandemic has shed light on the importance of coordination and collaboration among countries and stakeholders in different multilateral and global initiatives.

More efforts will be needed to encourage research and the use of technology to support policy making and provide evidence-based solutions. As health workforces show their crucial roles in managing public health emergencies like the COVID-19 pandemic, more efforts are needed to build their capacities and provide them with better working environments and motivating policies.

Following COVID-19, one can expect many changes in our daily life and activities. Although we are not sure about the *new normal* after COVID-19, we are sure about the fact that our lives and public health will not be the same as before. For agencies, systems, and individuals to survive after COVID-19, they must be resilient enough to cope with any expected changes.

## Abbreviations

MOH: ministries of health
NGO: nongovernmental organization
PHC: primary health care
RRT: rapid response team
SDG: Sustainable Development Goal
WHO: World Health Organization
